# Longitudinal evolution and risk profiles of comorbidity among people with HIV in China: a retrospective cohort study

**DOI:** 10.3389/fpubh.2025.1753514

**Published:** 2026-01-15

**Authors:** Chenye Liu, Liqin Sun, Xi Xiao, Yun He, Fang Zhao, Yinsong Luo, Dian Zhao, Yuxin Jiang, Kaiping Gao, Weijie Gong, Xiaorui Li, Tianqi Kong, Hongzhou Lu, Jiaye Liu

**Affiliations:** 1 School of Public Health, Shenzhen University Medical School, Shenzhen, Guangdong, China; 2 Department of Infectious Diseases, National Clinical Research Center for Infectious Diseases, Shenzhen Third People’s Hospital, Shenzhen, Guangdong, China

**Keywords:** cohort study, comorbidity, HIV, multimorbidity, PWH

## Abstract

**Objective:**

With increased longevity among people with HIV (PWH), multimorbidity has become a major challenge, but its long-term evolution remains poorly described. We aimed to characterize the longitudinal evolution of comorbidity and identify risk profiles for metabolic and mixed infectious–non-infectious multimorbidity among PWH initiating antiretroviral therapy in China.

**Methods:**

We conducted a retrospective cohort study of 5,950 PWH initiating antiretroviral therapy (ART) in Shenzhen, China (2009–2016), with follow-up through 2024. Twenty-six predefined comorbid conditions were classified as curable or incurable. Multimorbidity was defined as ≥2 conditions and grouped into six mutually exclusive comorbidity states. Evolution over 8 years was described using Sankey diagrams and stratified analyses. Multivariable Cox and logistic regression models were used to identify risk factors for incident metabolic multimorbidity and mixed infectious–non-infectious multimorbidity.

**Results:**

At ART initiation, 25.9% of participants had multimorbidity; by year 8 this had increased to 42.5%, mainly due to incurable multimorbidity (7.7 to 25.5%). Among 1,464 participants free of all 26 conditions at baseline, 36.1% developed multimorbidity. Younger PWH (≤ 32 years) experienced a steeper decline in comorbidity-free status, whereas older participants (≥46 years) were more likely to accumulate incurable multimorbidity. Dyslipidemia was the earliest and most persistent comorbidity and formed the core of the most frequent multimorbidity clusters. In the metabolic model, older age (≥46 vs. ≤ 25 years; hazard ratio [HR] 3.42, 95% confidence interval [CI] 2.60–4.49), higher body mass index (BMI), male sex, elevated fasting glucose, non-nucleoside reverse transcriptase inhibitor (NNRTI)-based regimen and reduced estimated glomerular filtration rate (eGFR) were associated with increased risk. For mixed infectious–non-infectious multimorbidity, male sex, injection drug use (IDU), low CD4 count, thrombocytopenia and opportunistic infections were risk factors, whereas higher BMI and being married were protective.

**Conclusion:**

Among relatively young Chinese PWH, multimorbidity increases rapidly after ART initiation, driven by metabolic and other incurable conditions centered on dyslipidemia. Distinct risk profiles for metabolic and mixed infectious–non-infectious patterns support integrating early cardiometabolic screening, intensifying infection control and proactive management of dyslipidemia into routine HIV care.

## Introduction

With the widespread adoption and ongoing optimization of antiretroviral therapy (ART), the life expectancy of people with HIV (PWH) has markedly increased (1–3). However, this success has introduced new challenges. PWH are now developing chronic comorbidities at younger ages than their HIV-negative counterparts, making multimorbidity a growing concern (2). Multimorbidity is conventionally defined as the simultaneous or sequential presence of two or more chronic health conditions within a given timeframe (4, 5). In the context of HIV, multimorbidity generally refers to the coexistence of at least two conditions other than HIV infection itself; in this study, we operationally included both chronic non-communicable diseases and chronic or recurrent infections in this definition. The combined effects of chronic HIV-related inflammation, immune dysregulation, and long-term exposure to antiretroviral therapy (ART) may shape comorbidity states in PWH that differ from those observed in the general population (2, 5, 6).

In addition to an increased risk of non-communicable diseases (NCDs)—such as hypertension, diabetes, and dyslipidemia—PWH also face a persistently high burden of chronic or recurrent infections, including hepatitis B, syphilis, and various opportunistic infections. These infections arise from ongoing immune dysfunction, high-risk behaviors, and complex interactions between pathogen latency, immune activation, and systemic metabolic dysregulation. Unlike in the general population, where multimorbidity typically emerges at older ages, PWH often experience multiple comorbidities at a younger age (6, 7). Moreover, the prevalence of multimorbidity rises steadily with age (8), underscoring the importance of investigating multimorbidity not only in older but also in younger PWH.

Although several large cohort studies have provided valuable insights into the burden and risk factors of multimorbidity among PWH (9, 10), much of the literature remains cross-sectional, focusing on a limited number of conditions. Even longitudinal studies have typically examined incidence and prevalence of comorbidities, but have rarely described how comorbidity states evolve over longer periods in routine HIV care (8, 11). As HIV care increasingly shifts from a focus on viral suppression to lifelong chronic disease management, a deeper understanding of multimorbidity dynamics is urgently needed.

In this study, we aimed to characterize the longitudinal evolution of comorbidity states over an 8-year period in a cohort of PWH in Shenzhen, China. We further identified the most prevalent and clinically significant multimorbidity clusters, described the evolution of multimorbidity, and examined risk factors associated with the development of metabolic-related and mixed infectious–non-infectious multimorbidity patterns.

## Methods

### Study design and population

We conducted a retrospective cohort study at the Third People’s Hospital of Shenzhen, the designated hospital for HIV care in Shenzhen, China. The cohort comprises approximately 22,000 PWH who have received ART at this hospital since 2009. ART is provided free-of-charge in accordance with national HIV treatment guidelines. PWH attend routine follow-up visits approximately every 3 months for continuous ART prescriptions and health monitoring. Detailed baseline and follow-up assessments, including demographic characteristics, clinical diagnoses, and laboratory measurements, are collected as part of routine clinical care and recorded in the hospital’s electronic medical record system. Further details on cohort characteristics, including prior studies using this dataset, can be found in the Supplementary materials (Brief Introduction of the Cohort).

Participants were eligible if they had a confirmed HIV diagnosis, initiated ART between January 1, 2009, and June 30, 2016, and had follow-up data available through June 30, 2024. Individuals with missing ART initiation dates or fewer than three recorded diagnostic encounters were excluded to reduce potential bias.

### Disease ascertainment and classification

All clinical outcomes were identified using ICD-10 diagnostic codes and clinician-documented diagnoses recorded in the hospital electronic medical records system at the Third People’s Hospital of Shenzhen, with confirmation based on relevant laboratory, imaging, and pathology findings in accordance with standardized diagnostic guidelines. A total of 26 conditions were selected for inclusion in the analysis. These conditions were chosen based on their prevalence in the general population and clinical relevance among PWH. The selected conditions included: (1) Hypertension, (2) Diabetes mellitus, (3) Dyslipidemia, (4) Cardiovascular disease (CVD), (5) Chronic obstructive pulmonary disease (COPD), emphysema, or chronic bronchitis, (6) Esophageal, gastric, or duodenal diseases, (7) Hepatitis B virus (HBV) infection, (8) Hepatitis C virus (HCV) infection, (9) Chronic kidney disease (CKD), (10) Non-AIDS-related cancers, (11) Osteoporosis, (12) Mental health disorders, (13) Sleep disorders, (14) Syphilis, (15) Human papillomavirus (HPV) infection, (16) Metabolic dysfunction–associated steatotic liver disease (MASLD), (17) AIDS-defining cancers, (18) HIV-associated encephalopathy, (19) Pneumocystis jirovecii pneumonia (PJP), (20) Nontuberculous mycobacterial infections, (21) Cytomegalovirus (CMV) infection, (22) Herpes simplex virus (HSV) infection, (23) Varicella-zoster virus (VZV) infection, (24) Toxoplasmosis, (25) Fungal infections, (26) Tuberculosis (TB). Full details on disease definitions and classification are available in the Supplementary materials.

### Follow-up and evolution of comorbidity states

ART initiation was defined as day zero, and a 90-day landmark period was applied to reduce misclassification of pre-existing comorbidities (12). Any predefined comorbid condition diagnosed on or before day 90 relative to ART initiation, including those documented prior to ART initiation, was treated as a baseline condition. The follow-up period was subsequently divided into three distinct phases: baseline (on or before day 90 after ART initiation), Wave1 (day 90 to 4 years), and Wave2 (4 to 8 years).

To characterize multimorbidity dynamics, we operationally classified the 26 predefined conditions into curable and incurable categories. This classification was informed by established disease-specific clinical guidelines and the typical clinical trajectory of each condition, as summarized in Supplementary Tables 1–3. Conditions were classified as curable if they are generally reversible with appropriate therapy and do not require lifelong disease monitoring once resolved (e.g., syphilis, tuberculosis, HCV infection after direct-acting antiviral treatment, acute viral infections, and other transient or episodic infectious conditions). In contrast, conditions were categorized as incurable if they typically reflect chronic or progressive organ dysfunction requiring sustained or lifelong clinical surveillance and/or pharmacological management, even when well controlled (e.g., hypertension, diabetes, dyslipidemia, CKD, chronic HBV infection, osteoporosis, and cancers). This pragmatic, guideline-informed classification reflects the expected long-term disease course rather than short-term clinical fluctuations and is aligned with contemporary clinical guidelines and expert consensus applied in routine HIV care.

Based on this distinction and the number of coexisting conditions, the 26 predefined conditions were pragmatically grouped into six mutually exclusive comorbidity states for the evolution plots: incurable multimorbidity (≥2 incurable conditions), curable multimorbidity (≥2 curable conditions), mixed curable–incurable multimorbidity (coexistence of at least one incurable and one curable condition), single incurable disease, single curable disease, and no disease. Curable conditions were modelled as resolving events and were therefore allowed to appear and disappear across periods, whereas incurable conditions were treated as long-term diagnoses. For each follow-up period, multimorbidity states were defined based on all curable and incurable conditions recorded in that period.

In addition to these six curability-based states, we further defined two etiologically distinct multimorbidity patterns. Metabolic multimorbidity was defined as the presence of at least two metabolic conditions, including hypertension, diabetes mellitus, dyslipidaemia, MASLD, or osteoporosis. Mixed infectious–non-infectious multimorbidity was defined as the coexistence of at least one infectious and one non-infectious condition during the follow-up period. Infectious conditions included chronic viral hepatitis (HBV/HCV), TB, STIs (syphilis/HPV), and major opportunistic infections. A detailed classification of all 26 conditions is provided in Supplementary Table 3.

Loss to follow-up was defined as having no clinical encounter, laboratory test, or recorded death for more than 180 days before the last observed date in the cohort. For the Sankey diagrams, because each participant could occupy only one state per period, we applied a hierarchical assignment rule: within a given period, individuals whose follow-up ended more than 180 days before the end of that period and who did not develop any new diagnoses among the 26 predefined conditions during that period were allocated to a combined “dropout” state, whereas those who developed at least one new diagnosis were classified into the corresponding module.

### Covariates

We extracted key covariates from the hospital records and laboratory results. Demographic variables included age at diagnosis, sex, and marital status. Age at diagnosis was categorized as ≤ 25, 26–35, 36–45, and ≥46 years, approximating quartiles of the distribution and reflecting early, middle, and later adulthood among PWH in this cohort. Anthropometric and laboratory parameters included body mass index (BMI), high-density lipoprotein cholesterol (HDL-C), low-density lipoprotein cholesterol (LDL-C), fasting glucose (FPG), platelet count (PLT), alanine aminotransferase (ALT), and estimated glomerular filtration rate (eGFR), all obtained through standardized clinical protocols. HIV-related factors comprised baseline CD4 count, baseline ART regimen, route of HIV transmission, diagnosis-to-ART initiation interval, and the presence of opportunistic infections at baseline. Behavioral characteristics included smoking and alcohol use.

### Statistical analysis

All analyses were performed using R version 4.4.1 and RStudio version 2024.12.0. Descriptive statistics were used to summarize participant characteristics and the distribution of multimorbidity states across waves.

Sankey diagrams were generated using the ggsankey, ggplot2, and dplyr packages in R to visualize transitions in comorbidity states over time. Retrospective Sankey plots were also constructed to trace prior disease profiles among individuals who developed incurable or mixed curable–incurable multimorbidity patterns.

Stacked bar charts were drawn to compare the distribution of comorbidity states by sex and age groups across waves. For stratified descriptive analyses, age at diagnosis was additionally dichotomised at the cohort median age of 32 years. Differences between subgroups were assessed using Chi-square or Fisher’s exact tests.

Regression analyses focused on two common and clinically important patterns: metabolic multimorbidity and mixed infectious–non-infectious multimorbidity; participants with the corresponding pattern at baseline were excluded. Age was defined as age at HIV diagnosis and treated as a fixed baseline covariate in all regression models. Metabolic multimorbidity was modelled using Cox proportional hazards regression for time to first onset, whereas the mixed pattern was analysed using multivariable logistic regression for ever-occurrence over 8 years, because infectious conditions may resolve and recur, precluding a precise onset time for a sustained mixed state. For the Cox regression models, the proportional hazards assumption was assessed using Schoenfeld residuals. Adjusted hazard ratios (HRs) and odds ratios (ORs) with 95% confidence intervals (CIs) were reported, and *p*-values < 0.05 were considered statistically significant.

As sensitivity analyses, we repeated the Cox regression for metabolic-related multimorbidity using an extended 6-month ART initiation window and conducted wave-specific analyses restricted to Wave1 (day 90 to 4 years) using the same covariate set. Results are presented in Supplementary Figures 1–3.

## Results

### Baseline characteristics of participants

A total of 5,950 participants were included in the final analysis. At enrollment, 25.9% of participants had multimorbidity (Table 1). Compared with those without multimorbidity, participants with multimorbidity were older (median age: 35.0 vs. 31.0 years; interquartile range [IQR]: 29–43 vs. 27–38 years; *p* < 0.05), and more likely to be male (90.5% vs. 85.6%; *p* < 0.05). Significant differences in BMI were observed between the two groups (p < 0.05), with a higher proportion of underweight individuals among those with multimorbidity. Marital status also showed a significant difference between groups (*p* < 0.05), with a higher proportion of divorced, separated, or widowed individuals in the multimorbidity group (12.3% vs. 8.0%). Unhealthy lifestyle behaviors, including smoking and alcohol use, were more common in the multimorbidity group (all *p* < 0.05). The prevalence of hypertension, diabetes, and hypercholesterolemia was significantly higher among participants with multimorbidity (all *p* < 0.05). Additionally, these participants had lower baseline CD4 cell counts, higher HIV RNA levels, and a greater prevalence of HBV or HCV co-infection (all *p* < 0.05).

**Table 1:** Baseline characteristics of study participants according to multimorbidity status.

Characteristics	Total	No multimorbidity	Multimorbidity	*p*-value
*N*	5,950	4,411	1,539	
*Age at diagnosis, years*				<0.001
≤ 25	1,036 (17.4)	852 (19.3)	184 (12.0)	
26–35	2,750 (46.2)	2,128 (48.2)	622 (40.4)	
36–45	1,449 (24.4)	993 (22.5)	456 (29.6)	
≥46	715 (12.0)	438 (10.0)	277 (18.0)	
*Sex*				
Male	5,171 (86.9)	3,778 (85.6)	1,393 (90.5)	<0.001
Female	779 (13.1)	633 (14.4)	146 (9.5)	
*BMI, kg/m^2^*				
<18.5	930 (15.6)	647 (14.7)	283 (18.4)	0.001
18.5–23.9	4,039 (67.9)	3,052 (69.2)	987 (64.1)	
≥24	981 (16.5)	712 (16.1)	269 (17.5)	
*Marital status*				
Single	3,181 (53.5)	2,441 (55.3)	740 (48.1)	<0.001
Married	2,229 (37.5)	1,619 (36.7)	610 (39.6)	
Divorced or widowed	540 (9.1)	351 (8.0)	189 (12.3)	
*Route of HIV transmission*				
MSM	3,518 (59.1)	2,629 (59.6)	889 (57.8)	0.049
Heterosexual	2,201 (37.0)	1,619 (36.7)	582 (37.8)	
IDU	74 (1.2)	45 (1.0)	29 (1.9)	
Others	157 (2.6)	118 (2.7)	39 (2.5)	
*Baseline CD4 count, cells/μL*				<0.001
<200	2,137 (35.9)	1,437 (32.6)	700 (45.5)	
200–349	2,484 (41.7)	1960 (44.4)	524 (34.0)	
350–499	1,006 (16.9)	768 (17.4)	238 (15.5)	
≥500	323 (5.4)	246 (5.6)	77 (5.0)	
*HIV RNA, copies/mL*				<0.001
<5,000	524 (8.8)	433 (9.8)	91 (5.9)	
5,000–99,999	3,538 (59.5)	2,454 (55.6)	1,084 (70.4)	
≥100,000	1888 (31.7)	1,524 (34.5)	364 (23.7)	
Smoking	1,292 (21.7)	918 (20.8)	374 (24.3)	0.003
Drinking	1,409 (23.7)	1,015 (23.0)	394 (25.6)	0.045
Hypertension	82 (1.4)	6 (0.1)	76 (4.9)	<0.001
Diabetes	219 (3.7)	18 (0.4)	201 (13.1)	<0.001
Hypercholesterolemia	4,056 (68.2)	2,645 (60.0)	1,411 (91.7)	<0.001
FPG, mmol/L	5.01 (4.7–5.4)	5.0 (4.7–5.3)	5.0 (4.7–5.5)	<0.001
HDL-C, mg/dL	48.7 (41.4–56.5)	49.1 (41.8–56.8)	48.0 (40.0–55.3)	<0.001
LDL-C, mg/dL	94.0 (78.5–111.8)	93.2 (78.5–111.0)	95.9 (79.7–114.5)	0.005
TG, mg/dL	113.3 (80.6–167.4)	105.4 (76.2–156.7)	135.5 (93.9–193.0)	<0.001
TC, mg/dL	162.0 (141.5–185.6)	161.3 (141.9–184.3)	163.6 (141.2–188.5)	0.156
TyG index	8.5 (8.2–9.0)	8.5 (8.1–8.9)	8.8 (8.4–9.1)	<0.001
Cr, mg/dL	0.8 (0.7–0.9)	0.8 (0.7–0.9)	0.8 (0.7–0.9)	<0.001
AST, U/L				<0.001
Normal	5,422 (91.1)	4,113 (93.2)	1,309 (85.1)	
≥1 × ULN	406 (6.8)	244 (5.5)	162 (10.5)	
≥2 × ULN	122 (2.1)	54 (1.2)	68 (4.4)	
ALT, U/L				<0.001
Normal	5,093 (85.6)	3,865 (87.6)	1,228 (79.8)	
≥1 × ULN	646 (10.9)	420 (9.5)	226 (14.7)	
≥2 × ULN	211 (3.5)	126 (2.9)	85 (5.5)	
WBC, 10^9/L	5.2 (4.3–6.3)	5.2 (4.3–6.3)	5.2 (4.2–6.4)	0.463
PLT, 10^9/L	201.0 (167.0–240.0)	201.0 (168.0–237.0)	203.0 (164.0–249.0)	0.219
HBV	327 (5.5)	52 (1.2)	275 (17.9)	<0.001
HCV	67 (1.1)	6 (0.1)	61 (4)	<0.001

Continuous variables are presented as median (IQR: 25th–75th percentile) and were compared using the Mann–Whitney *U*-test, and categorical variables are presented as frequency (%) and were compared using Pearson’s *χ*^2^ test. BMI, body mass index; HIV, human immunodeficiency virus; MSM, men who have sex with men; IDU, injection drug use; FPG, fasting plasma glucose; HDL-C, high-density lipoprotein cholesterol; LDL-C, low-density lipoprotein cholesterol; TG, triglycerides; TC, total cholesterol; TyG index, triglyceride–glucose index (calculated as ln [fasting triglycerides (mg/dL) × fasting glucose (mg/dL) ÷ 2]); Cr, serum creatinine; AST, aspartate aminotransferase; ALT, alanine aminotransferase; ULN, upper limit of normal (ALT ULN: 30 U/L for males and 19 U/L for females; AST ULN: 35 U/L for males and 31 U/L for females); WBC, white blood cells; PLT, platelet count; HBV, hepatitis B virus; HCV, hepatitis C virus; CD4, cluster of differentiation 4; HIV RNA, human immunodeficiency virus ribonucleic acid.

### Evolution of comorbidity states during 8-year follow-up in PWH

**Figure 1:**
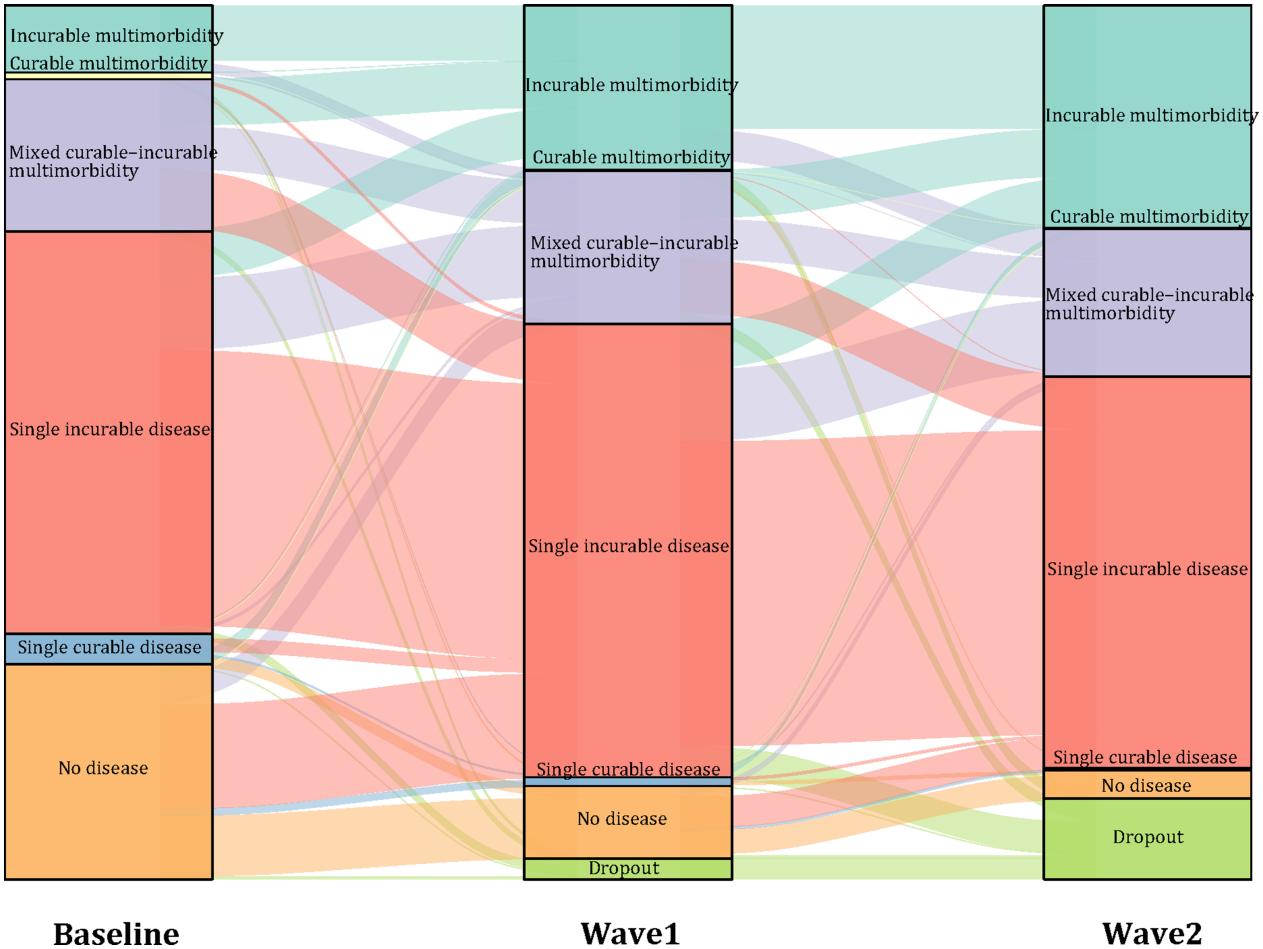
Evolution of PWH across six comorbidity states and dropout to follow-up during three time periods over an 8-year follow-up (*N* = 5,950). The height of the boxes and the thickness of the stripes are proportional to the amount of PWH belonging to the pattern and moving from the pattern, respectively.

Over the 8-year follow-up period, the distribution of comorbidity states among PWH exhibited notable temporal shifts. At baseline, most participants were classified as having either no comorbidity (24.61%) or a single incurable condition (46.03%) among the 5,950 individuals included. The overall prevalence of multimorbidity, including incurable, curable, and mixed patterns, increased from 25.87% at baseline to 42.49% by Wave2 (Figure 1, Supplementary Table 4). The most substantial increase was observed in the incurable multimorbidity pattern, which rose from 7.66% at baseline to 18.82% in Wave1, and further to 25.50% by Wave2, representing a more than threefold increase. The mixed curable–incurable multimorbidity pattern remained relatively stable over time, consistently affecting approximately 1,000 participants (16–18% of the cohort). In contrast, the curable multimorbidity and single curable disease patterns steadily declined, with their combined prevalence decreasing from 4.27% to just 0.3%.

Among the 1,464 participants who were free of all 26 predefined conditions at baseline, 528 (36.07%) developed multimorbidity during the follow-up period. Of these, 277 transitioned during Wave1, and an additional 251 during Wave2.

### Distribution of comorbidity states by sex and age

**Figure 2:**
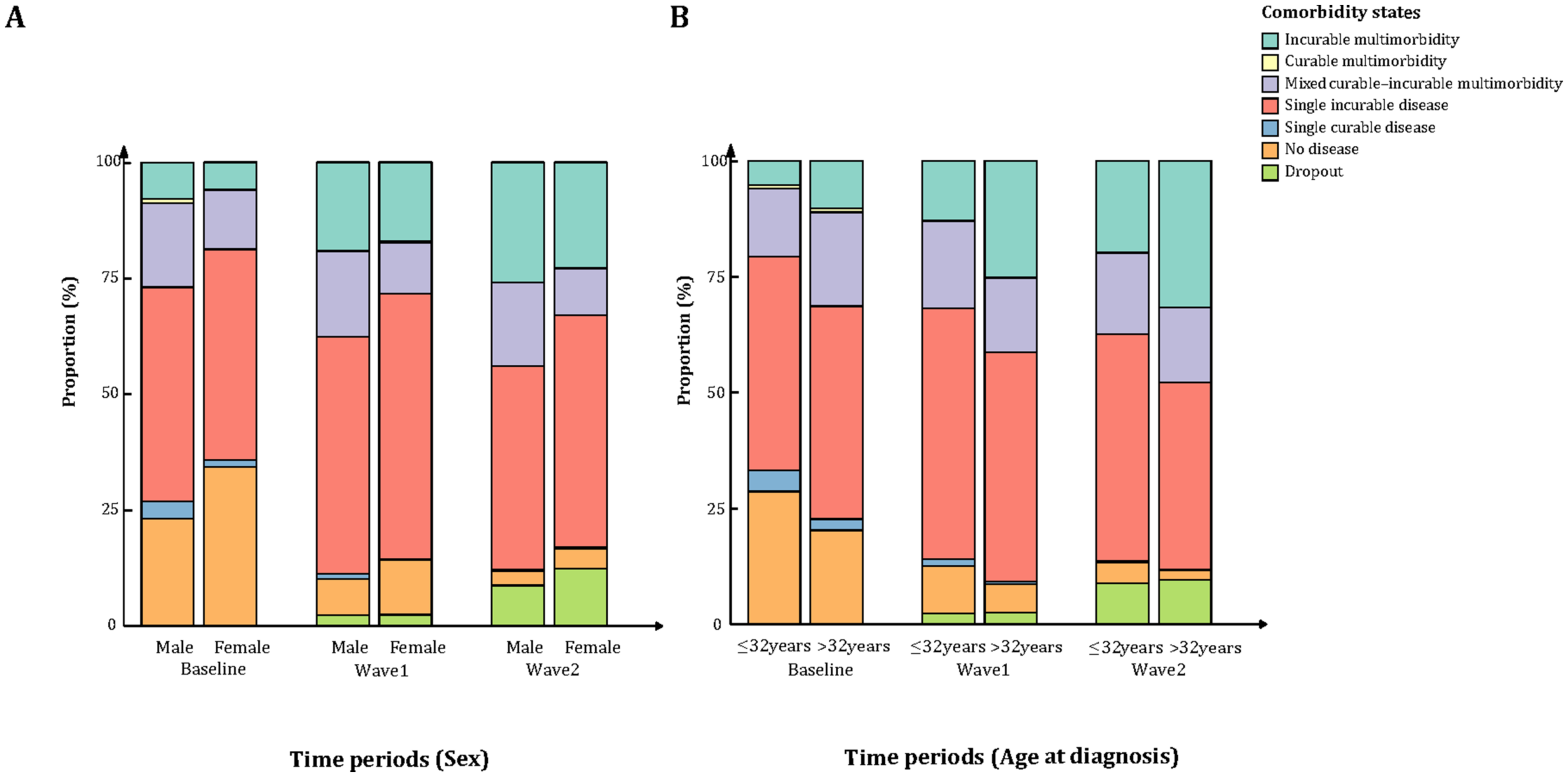
Distribution of comorbidity states by sex and age across different time periods. **(A)** Proportions of comorbidity states in different time periods stratified by sex. **(B)** Proportions of comorbidity states in different time periods stratified by age at diagnosis.

In the sex-stratified analysis, females consistently exhibited a lower prevalence of multimorbidity across all waves compared to males. The proportion of participants without any comorbidity declined markedly in both sexes, with a sharper decrease observed in females (from 34.27 to 4.36%) than in males (from 23.15 to 3.06%) (*p* < 0.05 for both). In contrast, males showed a greater increase in incurable multimorbidity over time, rising from 7.95 to 25.91%, compared with an increase from 5.78 to 22.72% in females (p < 0.05 for both). Meanwhile, the mixed curable–incurable multimorbidity pattern remained relatively stable in males (18.14 to 17.95%) but declined modestly in females (12.71 to 10.14%) (Figure 2A, Supplementary Tables 5–7).

Age-stratified analysis showed that individuals aged >32 years consistently had a higher burden of multimorbidity across all waves (Figure 2B, Supplementary Tables 8–10). At baseline, 31.36% of older participants had multimorbidity compared to 20.70% in those aged ≤32 years (*p* < 0.05). As follow-up progressed, multimorbidity increased significantly in both groups (*p* < 0.05), mainly driven by the rise in incurable multimorbidity. By Wave2, the prevalence of incurable multimorbidity reached 32.56% in the older group and 19.90% in the younger group (p < 0.05). While the older group initially had a higher prevalence of mixed multimorbidity (20.29% vs. 14.74%; *p* < 0.05), this difference diminished over time, and by Wave2 the proportions had converged (16.20% vs. 17.61%; *p* = 0.16). The proportion of participants with no comorbidity declined in both age groups, with a more substantial reduction among younger participants (from 28.66 to 4.37%; *p* < 0.05).

### Evolution and origins of top incurable and mixed curable–incurable multimorbidity patterns over time

**Figure 3:**
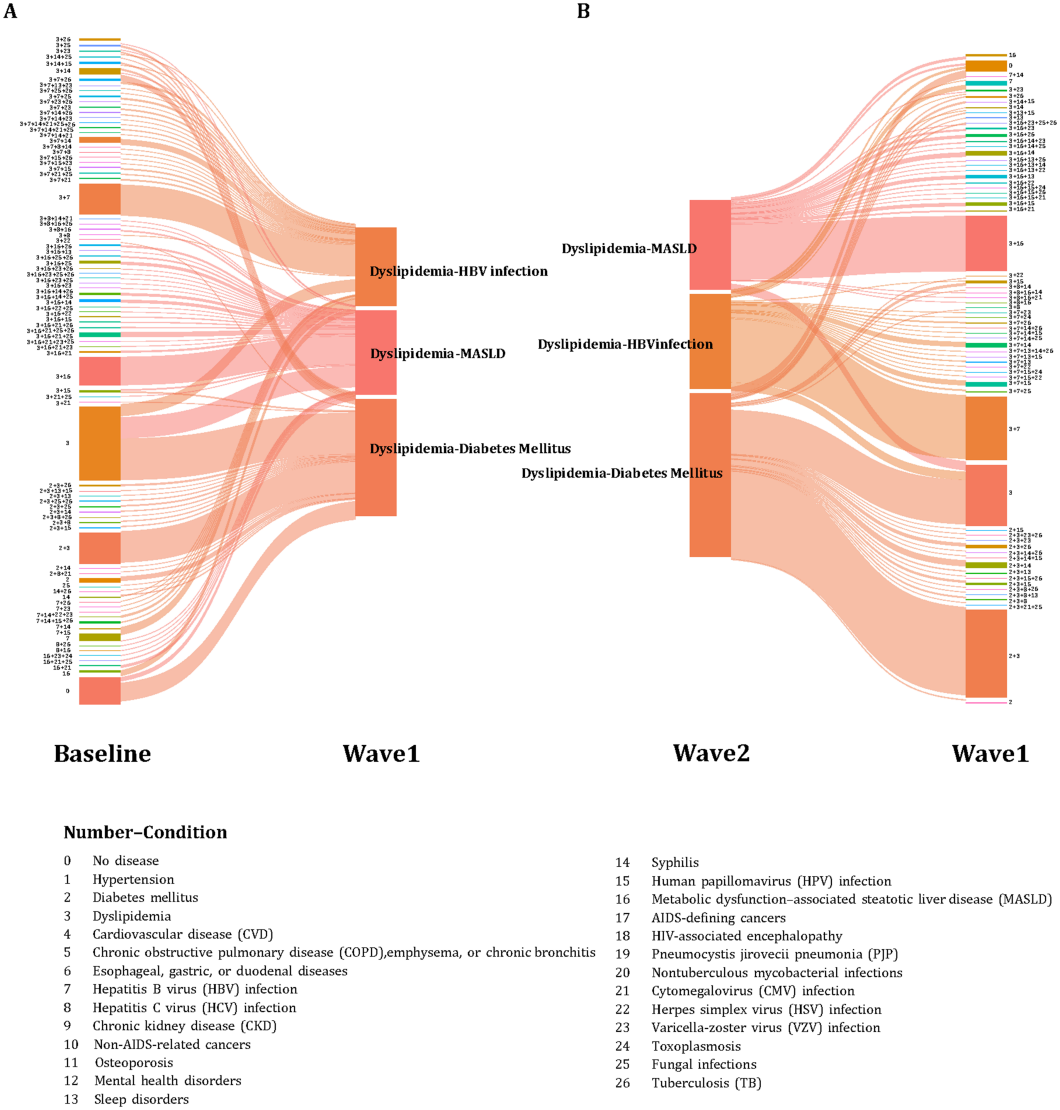
Retrospective Sankey diagrams illustrating the top three clusters’ transition for incurable multimorbidity pattern. Numbers nearby the clusters represent different 26 predefined conditions. **(A)** Transitions of Wave1’s top three clusters from baseline (*n* = 739). **(B)** Transitions of Wave2’s top three clusters from Wave1 (*n* = 916).

In the top three incurable multimorbidity clusters, most individuals maintained the same disease combinations through follow-up, reflecting considerable structural stability. Specifically, individuals classified under the “diabetes–dyslipidemia,” “dyslipidemia–HBV,” or “dyslipidemia–MASLD” at baseline, including those with coexisting curable diseases, largely remained within the same top three incurable disease clusters in Wave1. Within these groups, 18.00, 37.21, and 46.00% of individuals, respectively, had coexisting curable diseases, primarily infections. Among those with curable diseases at baseline, 22.22, 31.25, and 53.97% had two or more curable conditions, respectively. By Wave2, among individuals who remained in the same incurable multimorbidity clusters, the proportions with coexisting curable diseases had declined to 15.07, 20.10, and 27.86%, respectively (Figure 3).

**Figure 4:**
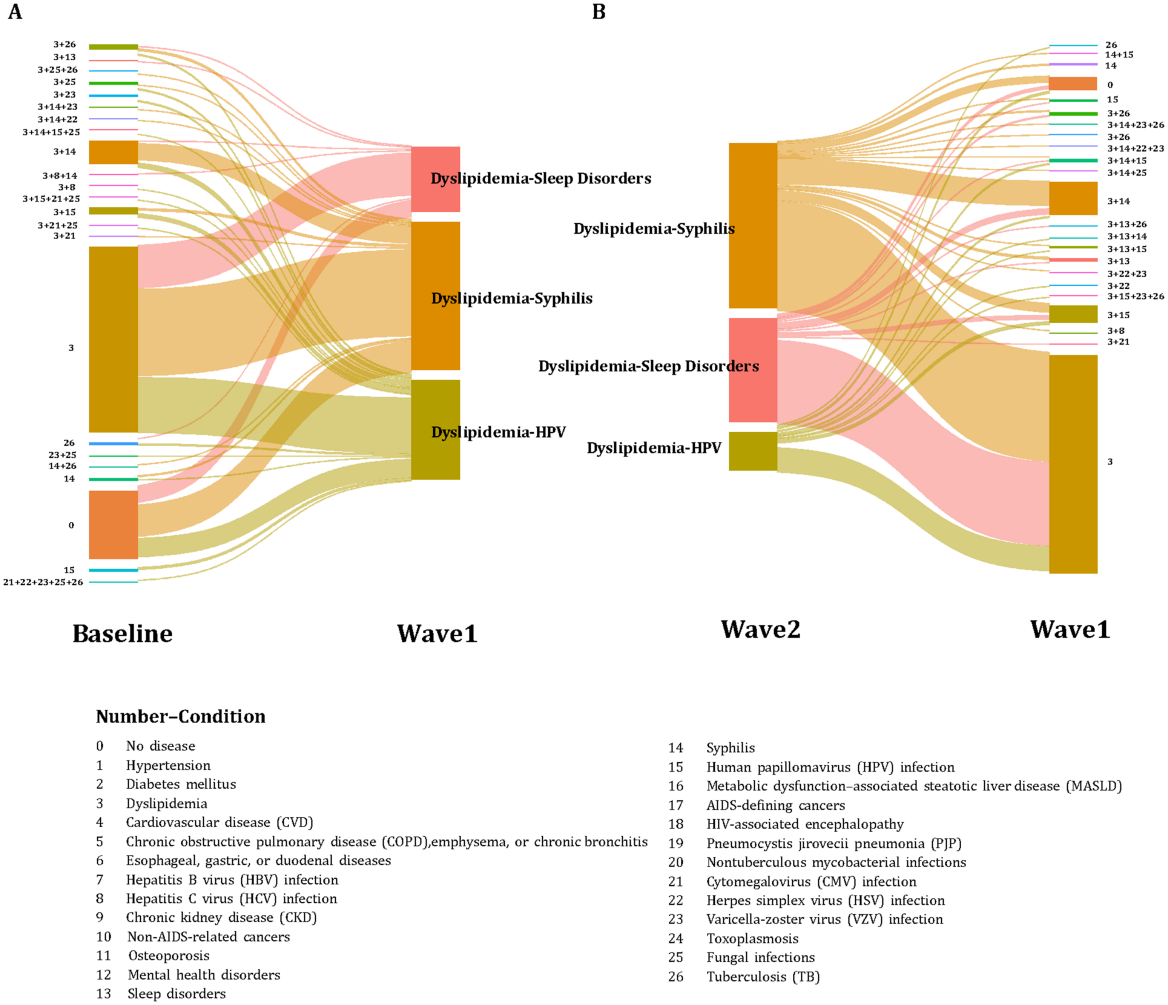
Retrospective Sankey diagrams illustrating the top three clusters’ transition for mixed curable–incurable multimorbidity pattern. Numbers nearby the clusters represent different 26 predefined conditions. **(A)** Transitions of wave 1’s top three clusters from baseline (*n* = 363). **(B)** Transitions of wave 2’s top three clusters from wave 1 (*n* = 359).

In the mixed curable–incurable pattern, more variation was observed. “Dyslipidemia–syphilis” remained the most common across waves (18.71, 16.51, and 19.17%), while “dyslipidemia–HPV” declined from 11.13% in Wave1 to 4.47% in Wave2. In contrast, “dyslipidemia–sleep disorders” increased to 12.02% in Wave2, becoming the second most frequent combination (Table 2, Figure 4).

**Table 2:** Distribution of the top three incurable and mixed curable–incurable multimorbidity patterns.

Time period	Incurable multimorbidity			Mixed curable–incurable multimorbidity		
	Pattern	N	Proportion, %	pattern	N	Proportion, %
Baseline	Total individuals	456		Total individuals	1,037	
	Diabetes and Dyslipidemia	115	25.22	Dyslipidemia and Syphilis	194	18.71
	Dyslipidemia and HBV	114	25.00	Dyslipidemia and TB	101	9.74
	Dyslipidemia and MASLD	98	21.49	Dyslipidemia and HPV	76	7.33
Wave1	Total individuals	1,143		Total individuals	1,042	
	Diabetes and Dyslipidemia	309	27.03	Dyslipidemia and Syphilis	172	16.51
	Dyslipidemia and MASLD	222	19.42	Dyslipidemia and HPV	116	11.13
	Dyslipidemia and HBV	208	18.20	Dyslipidemia and Sleep Disorders	75	7.20
Wave2	Total individuals	1,651		Total individuals	1,007	
	Diabetes and Dyslipidemia	431	26.11	Dyslipidemia and Syphilis	193	19.17
	Dyslipidemia and HBV	250	15.14	Dyslipidemia and Sleep Disorders	121	12.02
	Dyslipidemia and MASLD	235	14.23	Dyslipidemia and HPV	45	4.47

Percentages are calculated within each multimorbidity type (incurable vs mixed curable–incurable) and time period, using the total number of individuals with the corresponding multimorbidity type as the denominator. Only the three most frequent pairwise patterns are shown for each multimorbidity type and time period. HBV, hepatitis B virus; MASLD, metabolic dysfunction–associated steatotic liver disease; HPV, human papillomavirus; TB, tuberculosis.

### Distinct risk factors for metabolic versus infectious–non-infectious multimorbidity patterns

**Figure 5:**
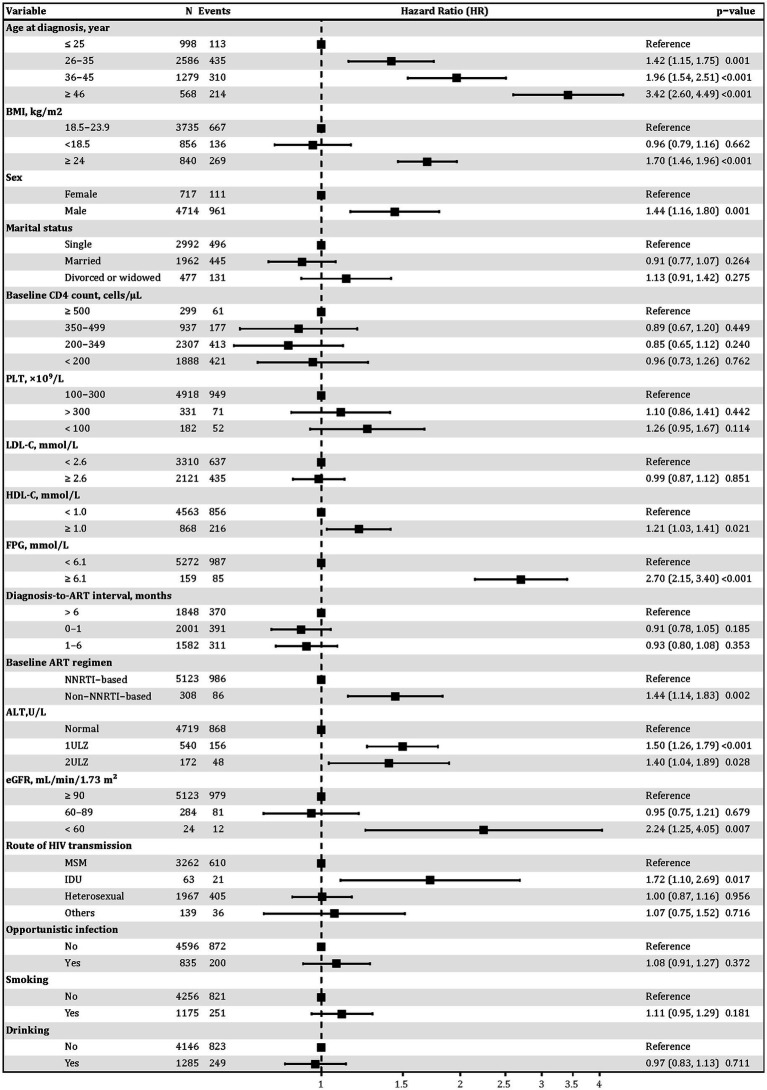
Forest plot of metabolic-related multimorbidity outcome derived from multivariable Cox regression. Adjusted hazard ratios (HRs) and 95% confidence intervals (CIs) are shown from Cox proportional hazards models including all covariates listed. BMI, body mass index; PLT, platelet count; LDL-C, low-density lipoprotein cholesterol; HDL-C, high-density lipoprotein cholesterol; FPG, fasting plasma glucose; ALT, alanine aminotransferase; eGFR, estimated glomerular filtration rate; MSM, men who have sex with men; IDU, injection drug use; HIV, human immunodeficiency virus; ULN, upper limit of normal; ART, antiretroviral therapy; CD4, cluster of differentiation 4; NNRTI, non-nucleoside reverse transcriptase inhibitor; HR, hazard ratio; CI, confidence interval.

In the metabolic multimorbidity model (Figure 5), older age was a strong predictor: individuals aged ≥46 had a 3.42-fold increased risk compared to those ≤25 (HR = 3.42, 95% CI: 2.60–4.49, *p* < 0.05). Overweight or obesity (BMI ≥ 24) was associated with higher risk (HR = 1.70, 95% CI: 1.46–1.96, *p* < 0.05), as was male sex (HR = 1.44, 95% CI: 1.16–1.80, *p* < 0.05). Elevated HDL-C (≥1.0 mmol/L) and FPG (≥6.1 mmol/L) were also significant (HR = 1.21, 95% CI: 1.03–1.41, *p* < 0.05; HR = 2.70, 95% CI: 2.15–3.40, *p* < 0.05). ALT ≥1 × ULN and ≥2 × ULN increased the risk by 50 and 40%, respectively (both *p* < 0.05), while reduced eGFR (<60 mL/min/1.73 m^2^) was associated with higher risk (HR = 2.24, 95% CI: 1.25–4.05, *p* < 0.05). Compared to those on non-nucleoside reverse transcriptase inhibitor (NNRTI)-based regimens, PWH on non-NNRTI regimens had a higher risk (HR = 1.44, 95% CI: 1.14–1.83, *p* < 0.05). Those with IDU also showed increased risk (HR = 1.72, 95% CI: 1.10–2.69, *p* < 0.05) compared to MSM. Baseline CD4 count, smoking, diagnosis-to-ART interval, and alcohol use were not significantly associated. No meaningful violations of the proportional hazards assumption were detected (Supplementary Table 11). Results were robust in sensitivity analyses, with consistent findings observed using a 6-month ART initiation window and in analyses restricted to Wave1 (Supplementary Figures 1, 2).

**Figure 6:**
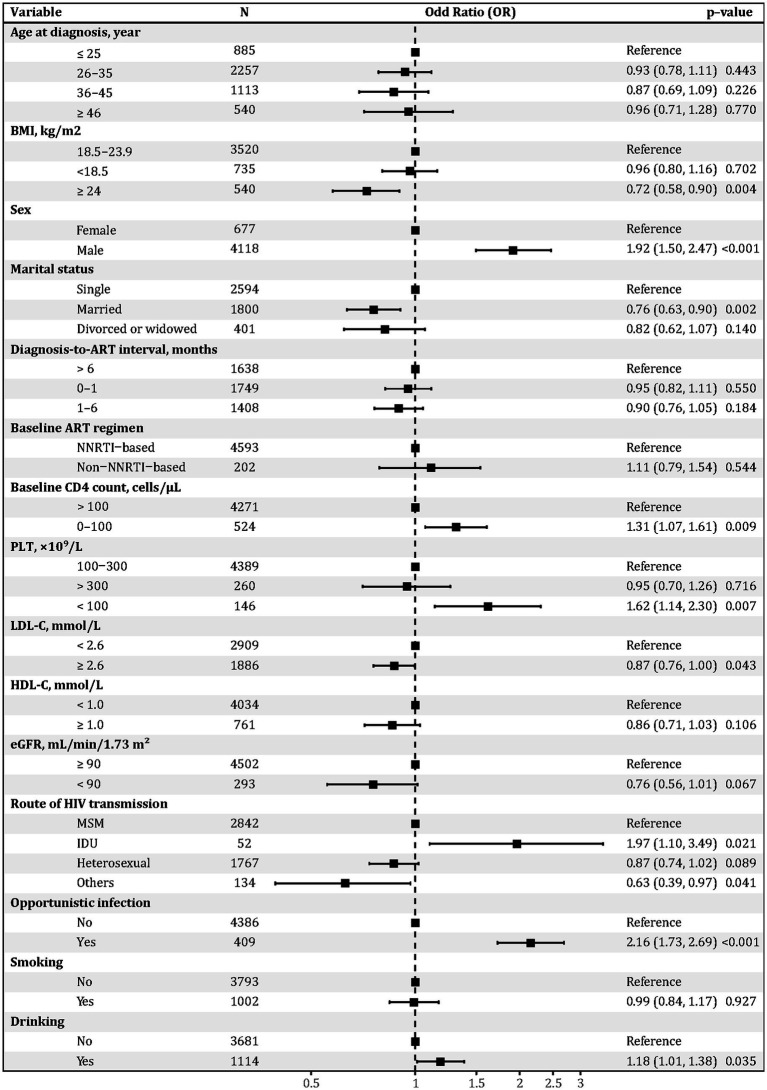
Forest plot of mixed infectious–non-infectious multimorbidity outcome derived from multivariable logistic regression. Adjusted odds ratios (ORs) and 95% confidence intervals (CIs) are shown from multivariable logistic regression models including all covariates listed. BMI, body mass index; PLT, platelet count; LDL-C, low-density lipoprotein cholesterol; HDL-C, high-density lipoprotein cholesterol; eGFR, estimated glomerular filtration rate; MSM, men who have sex with men; IDU, injection drug use; HIV, human immunodeficiency virus; ART, antiretroviral therapy; NNRTI, non-nucleoside reverse transcriptase inhibitor; CD4, cluster of differentiation 4.

In the mixed infectious–non-infectious model (Figure 6), male sex (OR = 1.92, 95% CI: 1.50–2.47, *p* < 0.05) and IDU (OR = 1.97, 95% CI: 1.10–3.49, *p* < 0.05) were strong risk factors. In contrast, BMI ≥ 24 was protective (OR = 0.72, 95% CI: 0.58–0.90, *p* < 0.05), as was being married (OR = 0.76, 95% CI: 0.63–0.90, *p* < 0.05). CD4 ≤ 100 cells/μL (OR = 1.31, 95% CI: 1.07–1.61, *p* < 0.05), PLT < 100 × 10^9^/L (OR = 1.62, 95% CI: 1.14–2.30, *p* < 0.05), and a history of opportunistic infections (OR = 2.16, 95% CI: 1.73–2.69, *p* < 0.05) were all independently associated. Alcohol consumption was modestly associated (OR = 1.18, p < 0.05), while age, HDL-C, diagnosis-to-ART interval, baseline ART regimen, and eGFR were not significant in this model. Sensitivity analyses restricted to Wave1 yielded consistent results (Supplementary Figure 3).

## Discussion

This study presents an 8-year longitudinal cohort analysis examining the evolution and risk factors of multimorbidity among PWH in Shenzhen, China. Over the follow-up period, we observed a significant increase in multimorbidity prevalence. Notably, the prevalence of incurable multimorbidity nearly tripled and became the dominant pattern. Although males consistently exhibited higher multimorbidity burdens, females experienced a faster rate of increase, and younger individuals (≤32 years) showed steeper rises despite lower baseline burdens. Most multimorbidity patterns involved combinations of non-communicable diseases with curable infectious conditions, highlighting a dual burden of disease. Additionally, distinct risk profiles were identified for metabolic versus mixed infectious–non-infectious multimorbidity patterns. These findings underscore the necessity of continuous monitoring and targeted interventions tailored to specific multimorbidity trajectories, emphasizing the importance of personalized chronic disease management for PWH.

The sharp increase in multimorbidity, particularly incurable patterns, reflects a shifting disease landscape in the ART era. While the incidence of infectious comorbidities declined with prolonged ART, the burden of non-AIDS-related chronic conditions—such as dyslipidemia, diabetes, and hypertension—increased substantially, in line with previous findings (2). This transition may be driven by population aging (13), chronic immune activation (14–16), HIV-induced mitochondrial dysfunction (17–19), gut microbiota dysbiosis (20), and long-term ART-related toxicities (21, 22). Dyslipidemia emerged as a key factor in both incurable and mixed-curability patterns, likely due to HIV-related immune activation, ART-induced metabolic effects, and its impact on cardiovascular, liver, and glucose metabolism (21, 23–27). Its frequent co-occurrence with both metabolic and infectious comorbidities underscores its potential as an early marker and modifiable entry point for multimorbidity surveillance and prevention. Collectively, these shifts call for integrated models of HIV care that anticipate evolving chronic disease risks and adapt accordingly across the treatment continuum.

Subgroup analyses revealed sex- and age-related disparities in multimorbidity trends. Although men consistently bore a greater burden of multimorbidity, women experienced a steeper increase over time. This may reflect sex-based immunological differences as well as gender-specific barriers to health service access, delayed diagnosis, or reduced screening uptake (7, 28, 29). Age was another consistent predictor of multimorbidity; older PWH had significantly greater burdens across all patterns, corroborating prior cohort studies (8). Notably, despite a relatively young mean age of just over 30 years in this cohort, a substantial multimorbidity burden was already present at baseline, and younger individuals (≤32 years) showed a faster decline in comorbidity-free status over time. This accelerated accumulation may reflect early onset of immunosenescence, chronic low-grade inflammation, and ART-related metabolic disturbances. It may also signal insufficient attention to routine screening and health maintenance in younger PWH (29, 30). These findings underscore the need to prioritize early screening and tailored interventions for older males, while also emphasizing the importance of proactive chronic disease surveillance among younger populations.

The contrasting risk profiles of metabolic versus infectious–non-infectious multimorbidity highlight important differences in etiology and management priorities. Metabolic multimorbidity was primarily associated with older age, overweight or obesity, elevated fasting glucose, and organ dysfunction, likely reflecting cumulative effects of ART toxicity, systemic inflammation, and metabolic derangements (31, 32). Interestingly, elevated HDL-C also conferred increased risk, echoing previous studies suggesting a potential U-shaped relationship with cardiovascular risk in PWH (33, 34). In contrast, higher BMI appeared protective in the mixed-infectious model, possibly due to improved immune reconstitution in individuals with greater baseline nutritional reserves (8, 35). In addition, non-NNRTI-based regimen was associated with higher risk of metabolic multimorbidity, possibly due to effects of other ART classes (36). Furthermore, PWH with a history of IDU faced substantially higher risks in both patterns, likely driven by higher rates of co-infections, lower ART adherence, and greater cumulative immune damage (37–40). These findings support the use of risk stratification tools and differentiated care pathways—metabolic screening and lifestyle modification for at-risk individuals, and early immune recovery and infection control for those with high behavioral vulnerability.

This study has several strengths. The extended 8-year follow-up allowed us to describe long-term changes in comorbidity states beyond what can be seen in cross-sectional analyses. By jointly considering both curable and incurable conditions, we characterised a broader spectrum of comorbidity, including combinations of infections and NCDs that are particularly relevant among PWH. This operational approach illustrates a pragmatic way to incorporate infectious comorbidities into multimorbidity assessments and may complement traditional multimorbidity research that has focused mainly on NCDs.

### Study limitations

Several limitations should be acknowledged. First, the study was conducted at a single HIV treatment center, which may limit the generalizability of findings to other regions or healthcare settings. Second, despite efforts to standardize diagnoses using laboratory data, underdiagnosis or underdocumentation of conditions such as mental health disorders and behavioral diseases may persist. Third, age was defined at HIV diagnosis and modelled as a fixed baseline covariate; therefore, age-stratified results reflect baseline differences associated with baseline age rather than changes in attained age during follow-up. Finally, the distinction of curable and incurable was a pragmatic operationalisation and could not account for within-disease variation in severity, control, or treatment response; these dimensions may differentially affect prognosis and should be explored in future work. Future multicenter studies with prospective designs and richer behavioral datasets are needed to validate and expand upon these findings. Because the evolution observed in this study is descriptive, future work will incorporate trajectory-modelling approaches to more rigorously characterise longitudinal patterns.

## Conclusion

This study reveals a rising burden of multimorbidity among PWH in the ART era, characterized by dynamic shifts from infectious conditions to metabolic and behavioral comorbidities. Distinct multimorbidity trajectories and risk profiles underscore the need for tailored prevention and management strategies, particularly among high-risk subgroups such as older males and those with a history of injecting drug. Moving forward, research should focus on elucidating the mechanisms underlying multimorbidity clustering, evaluating the effectiveness of early interventions, and building integrated care models that address both infectious and non-communicable diseases across the life course of PWH.

## Data Availability

The data analyzed in this study is subject to the following licenses/restrictions: the dataset contains sensitive patient-level clinical information and is therefore subject to privacy and ethical restrictions. Data cannot be publicly shared. Access to the dataset may be granted upon reasonable request to the corresponding author and requires approval from the Institutional Review Board of the Third People’s Hospital of Shenzhen. Requests to access these datasets should be directed to JL, liujiaye1984@163.com.
