# Supplementary material for: Longitudinal evolution and risk profiles of comorbidity among people with HIV in China: a retrospective cohort study
Source: Front Public Health. 2026 Jan 15;13:1753514. doi: 10.3389/fpubh.2025.1753514 (PMC12852350; doi:10.3389/fpubh.2025.1753514)
Supplement: Supplementary file 1 [file Supplementary_file_1.docx]

**Supplementary material**

1. **Supplemental Brief introduction of the Cohort** 2
2. **Supplemental Table 1.** Clinical and laboratory diagnostic criteria for 26 conditions in the study 4
3. **Supplemental Table 2.** Included ICD-10 codes in each condition 9
4. **Supplemental Table 3.** Classification of the 26 conditions by curability, metabolic status, and infectious status 11
5. **Supplemental Table 4.** Distribution of six multimorbidity patterns and dropout across three waves 12
6. **Supplemental Table 5.** Comorbidity states distribution and sex difference in different waves 13
7. **Supplemental Table 6.** Comorbidity states change over time among male participants 15
8. **Supplemental Table 7.** Comorbidity states change over time among female participants 16
9. **Supplemental Table 8.** Comorbidity states distribution and diagnostic age difference in different waves 17
10. **Supplemental Table 9.** Comorbidity states change over time among younger (≤32 years) participants 19
11. **Supplemental Table 10.** Comorbidity states change over time among older (>32 years) participants 20
12. **Supplemental Table 11.** Schoenfeld residual–based tests of the proportional hazards (PH) assumption for the Cox model 21
13. **Supplemental Figure 1.** Sensitivity analysis of Cox regression on metabolic-related multimorbidity using an extended 6-month ART initiation window 21
14. **Supplemental Figure 2.** Sensitivity analysis of Cox regression on metabolic multimorbidity restricted to Wave1 23
15. **Supplemental Figure 3.** Sensitivity analysis of logistic regression on mixed infectious–non-infectious multimorbidity restricted to Wave1 24

**Supplemental Brief introduction of the Cohort**

This study is based on a cohort study at the Third People’s Hospital of Shenzhen, the only designated hospital for HIV care in Shenzhen, China. The hospital managed approximately 22,000 people with HIV (PWH) from 2009 to 2024. Antiretroviral therapy (ART) is provided free-of-charge according to national HIV treatment guidelines. PWH visit the hospital every three months for continuous ART prescriptions and health monitoring. Before starting ART, comprehensive baseline assessments are conducted, including HIV viral load, T-cell subsets, common opportunistic infections, complete blood count, liver and kidney function, thyroid function, electrocardiogram, bone density, and co-infections (e.g., CMV, HBV, HCV, syphilis, HPV). Detailed patient histories and lifestyle information are also collected. After initiating ART, patients are regularly followed up every three months with routine HIV viral load and T-cell subset testing, and additional tests as needed. The high reimbursement rate of Shenzhen’s health insurance policies contributes to a low loss-to-follow-up rate, providing a stable cohort and valuable clinical data for research.

The following are some studies we have conducted based on this cohort：

1. Li T, Sun L, He Y, et al. Increasing trends of overweight and obesity in treatment-naive people living with HIV in Shenzhen from 2014 to 2020: an emerging health concern. Front Public Health 2023; 11:1186838.
2. Sun L, He Y, Xu L, et al. Higher Risk of Dyslipidemia With Coformulated Elvitegravir, Cobicistat, Emtricitabine, and Tenofovir Alafenamide than Efavirenz, Lamivudine, and Tenofovir Disoproxil Fumarate Among Antiretroviral-Naive People Living With HIV in China. J Acquir Immune Defic Syndr 2022; 91: S8-S15.
3. Liu J, Hou Y, Sun L, et al. High population-attributable fractions of traditional risk factors for non-AIDS-defining diseases among people living with HIV in China: a cohort study. Emerging Microbes & Infections 2021; 10:416-423.
4. Liu J, Sun L, Hou Y, et al. Barriers to early diagnosis and treatment of persistently high burden of severely immunosuppressed patients with HIV-1: a quantitative and qualitative study. HIV Medicine 2020; 21:708-717.
5. Sun LQ, Liu JY, He Y, et al. Evolution of blood lipids and risk factors of dyslipidemia among people living with human immunodeficiency virus who had received first-line antiretroviral regimens for 3 years in Shenzhen. Chin Med J 2020; 133:2808-2815.
6. Li XR, Sun LQ, He Y, et al. HDL-C as a novel predictor of immune reconstitution in people living with HIV: insights from a baseline-to-dynamic change cohort study in China, 2005–2022. Frontiers in Immunology 2025; 16: 1520615.
7. Luo YS, Sun LQ, He Y, et al. The triglyceride-glucose index trajectories are associated with cardiovascular diseases in people living with HIV: evidence from a prospective cohort study in China, 2005–2022. BMC Public Health 2025; 25: 465.
8. Li X, Sun L, He Y, et al. Two-threshold defined immune non-responders are associated with long-term morbidity in people with HIV: a prospective cohort study. Emerg Microbes Infect. 2025;14(1):2539198.
9. Sun L, Zhu H, Rao M, et al. Increasing trends of hyperglycemia and diabetes in treatment-naive people living with HIV in Shenzhen from 2013 to 2019: An emerging health concern. Chin Med J (Engl). 2025;138(16):2043-2045.

**Supplemental Table 1. Clinical and laboratory diagnostic criteria for 26 conditions in the study**

| **Condition** | | **Clinical and laboratory diagnostic criteria** |
| --- | --- | --- |
| 1 | Hypertension [1] | Blood pressure ≥ 140/90 mmHg |
| 2 | Diabetes Mellitus [2] | HbA1c ≥ 6.5%, fasting plasma glucose ≥ 126 mg/dL, or use of antidiabetic medications, or a self-reported history of diabetes |
| 3 | Dyslipidemia [3] | TC ≥ 5.2 mmol/L, HDL-C ≤ 1.0 mmol/L, LDL-C ≥ 3.4 mmol/L, or TG ≥ 1.7 mmol/L |
| 4 | Cardiovascular Disease [4] | Confirmed by ECG, echocardiography, cardiac biomarkers, or coronary angiography |
| 5 | COPD (Chronic Obstructive Pulmonary Disease), Emphysema, Chronic Bronchitis [5] | FEV1/FVC < 0.70; use of anticholinergics |
| 6 | Diseases of Esophagus, Stomach, and Duodenum [6] | Diagnosis based on endoscopy, biopsy, or imaging |
| 7 | Hepatitis B virus (HBV) infection [7] | Persistent HBsAg positivity > 6 months |
| 8 | Hepatitis C virus (HCV) infection [8] | Positive anti-HCV and HCV RNA |
| 9 | Chronic kidney disease (CKD) [9] | Glomerular filtration rate <60 mL/min/1.73 m² for ≥ 3 months(assessed using the CKD-EPI equation) |
| 10 | Non–AIDS-related cancers [10] | Histopathological confirmation |
| 11 | Osteoporosis [11] | Bone mineral density T-score ≤ -2.5 |
| 12 | Mental health disorders [12] | Diagnosis per DSM-5 |
| 13 | Sleep disorders [13] | Diagnosis per ICSD-3 and PSG |
| 14 | Syphilis [14] | Positive RPR/VDRL and TPPA/FTA-ABS |
| 15 | Human papillomavirus (HPV) infection [15] | Positive high-risk HPV DNA |
| 16 | Metabolic dysfunction–associated steatotic liver disease (MASLD) [16] | Evidence of hepatic steatosis on imaging or histology in the absence of viral hepatitis and other specific causes, based on contemporary NAFLD/MASLD guidelines |
| 17 | AIDS-defining cancers [17] | Kaposi sarcoma, cervical cancer, or aggressive B-cell lymphoma |
| 18 | HIV-associated encephalopathy [18] | Cognitive decline in HIV+ after excluding other causes |
| 19 | Pneumocystis jirovecii pneumonia (PJP) [19] | Radiologic findings and P. jirovecii in BAL or sputum |
| 20 | Nontuberculous mycobacterial infections [20] | ≥ 2 positive sputum cultures and compatible imaging |
| 21 | Cytomegalovirus (CMV) infection [21] | CMV DNA PCR or inclusion bodies |
| 22 | Herpes simplex virus (HSV) infection [22] | Positive PCR or viral culture |
| 23 | Varicella-zoster virus (VZV) infection [23] | Clinical rash and positive PCR |
| 24 | Toxoplasmosis [24] | Positive IgM/IgG or CSF Toxoplasma DNA |
| 25 | Fungal infections [25] | Positive culture, microscopy, or serology (e.g., beta-D-glucan) |
| 26 | Tuberculosis (TB) [26] | AFB smear, culture, or PCR with radiographic evidence |

**Reference** (ordered according to the 26 diseases)：

1. Mancia G, Kreutz R, Brunström M, et al. 2023 ESH Guidelines for the management of arterial hypertension: The Task Force for the management of arterial hypertension of the European Society of Hypertension. J Hypertens. 2023;41(12):1874–2071. doi:10.1097/HJH.0000000000003480
2. American Diabetes Association. Standards of Medical Care in Diabetes—2024. Diabetes Care. 2024;47(Suppl 1):S1–S350. doi:10.2337/dc24-S001
3. Joint Committee for the Revision of Guidelines for the Management of Blood Lipids in China. Chinese guidelines for the management of blood lipids (2023 edition). Chinese Circulation Journal. 2023;38(3):237–271. (In Chinese). doi: 10.3969/j.issn.1000-3614.2023.03.001
4. Goff DC Jr, Lloyd-Jones DM, Bennett G, et al. ACC/AHA Guideline on the Assessment of Cardiovascular Risk. J Am Coll Cardiol. 2014;63(25 Pt B):2935–2959. doi:10.1016/j.jacc.2013.11.005
5. Global Initiative for Chronic Obstructive Lung Disease (GOLD). Global Strategy for the Diagnosis, Management, and Prevention of COPD – 2023 Report. Available at: https://goldcopd.org
6. Katz PO, Dunbar KB, Schnoll-Sussman FH, et al. ACG Clinical Guideline for the Diagnosis and Management of GERD. Am J Gastroenterol. 2022;117(1):27–56. doi:10.14309/ajg.0000000000001538
7. World Health Organization. Guidelines for the prevention, care and treatment of persons with chronic hepatitis B infection. WHO; 2015. Available at: [https://www.who.int/publications/i/item/9789241549059](https://www.who.int/publications/i/item/9789241549059)
8. Centers for Disease Control and Prevention (CDC). Hepatitis C Guidance: Testing, Management, and Treatment. Updated 2023. Available at: https://www.cdc.gov/hepatitis/hcv/index.htm
9. Kidney Disease: Improving Global Outcomes (KDIGO) CKD Work Group. KDIGO 2024 Clinical Practice Guideline for the Evaluation and Management of Chronic Kidney Disease. Kidney Int. 2024;105(4S):S117-S314. doi:10.1016/j.kint.2023.10.018
10. National Comprehensive Cancer Network (NCCN). NCCN Clinical Practice Guidelines in Oncology. Various editions by cancer type. Available at: https://www.nccn.org
11. Cosman F, de Beur SJ, LeBoff MS, et al. Clinician’s Guide to Prevention and Treatment of Osteoporosis. Osteoporos Int. 2014;25(10):2359–2381. doi:10.1007/s00198-014-2794-2
12. American Psychiatric Association. Diagnostic and Statistical Manual of Mental Disorders, Fifth Edition (DSM-5). American Psychiatric Publishing; 2013. Available at: https://www.psychiatry.org/psychiatrists/practice/dsm
13. American Academy of Sleep Medicine. International Classification of Sleep Disorders—Third Edition (ICSD-3). 2014. Available at: https://aasm.org/clinical-resources/international-classification-sleep-disorders/
14. Centers for Disease Control and Prevention. Sexually Transmitted Infections Treatment Guidelines, 2021. MMWR Recomm Rep. 2021;70(4):1–187. doi:10.15585/mmwr.rr7004a1
15. World Health Organization. WHO Guidelines for Screening and Treatment of Precancerous Lesions for Cervical Cancer Prevention. 2nd edition. 2021. Available at: [https://www.who.int/publications/i/item/9789240030824](https://www.who.int/publications/i/item/9789240030824)
16. Chalasani N, Younossi Z, Lavine JE, et al. The diagnosis and management of nonalcoholic fatty liver disease: Practice guidance from the American Association for the Study of Liver Diseases. Hepatology. 2018;67(1):328–357. doi:10.1002/hep.29367
17. Centers for Disease Control and Prevention. AIDS-Defining Conditions. Available at: https://www.cdc.gov/hiv/library/reports/hiv-surveillance/vol-32/content/appendix-c.html
18. Panel on Antiretroviral Guidelines for Adults and Adolescents. Guidelines for the Use of Antiretroviral Agents in Adults and Adolescents with HIV. U.S. Department of Health and Human Services. Updated May 13, 2025. Available at: [https://clinicalinfo.hiv.gov/en/guidelines/adult-and-adolescent-arv](https://clinicalinfo.hiv.gov/en/guidelines/adult-and-adolescent-arv).
19. Panel on Opportunistic Infections in Adults and Adolescents with HIV. Guidelines for the Prevention and Treatment of Opportunistic Infections. Updated 2022. Available at: https://clinicalinfo.hiv.gov
20. Daley CL, Iaccarino JM, Lange C, et al. Treatment of Nontuberculous Mycobacterial Pulmonary Disease: An Official ATS/ERS/ESCMID/IDSA Clinical Practice Guideline. Clin Infect Dis. 2020;71(4):e1–e36. doi:10.1093/cid/ciz236
21. Kotton CN, Kumar D, Caliendo AM, et al. The Third International Consensus Guidelines on the Management of Cytomegalovirus in Solid Organ Transplantation. Transplantation. 2018;102(6):900–931. doi:10.1097/TP.0000000000002191
22. Centers for Disease Control and Prevention. Sexually Transmitted Infections Treatment Guidelines, 2021. MMWR Recomm Rep. 2021;70(4):1–187. doi:10.15585/mmwr.rr7004a1
23. Centers for Disease Control and Prevention. Varicella-Zoster Virus Infection. Available at: https://www.cdc.gov/chickenpox
24. Centers for Disease Control and Prevention. Guidelines for the Prevention and Treatment of Opportunistic Infections in HIV-Infected Adults and Adolescents. Updated 2022.
25. Pappas PG, Kauffman CA, Andes DR, et al. Clinical Practice Guideline for the Management of Candidiasis: 2016 Update by the Infectious Diseases Society of America. Clin Infect Dis. 2016;62(4):e1–e50. doi:10.1093/cid/civ933
26. World Health Organization. WHO consolidated guidelines on tuberculosis. Module 3: Diagnosis – Rapid diagnostics for tuberculosis detection. 2022. Available at: [https://www.who.int/publications/i/item/9789240047327](https://www.who.int/publications/i/item/9789240047327)

**Supplemental Table 2. Included ICD-10 codes in each condition**

| **Condition** | | **ICD-10** |
| --- | --- | --- |
| 1 | Hypertension | I10-I15 |
| 2 | Diabetes Mellitus | E10-E14 |
| 3 | Dyslipidemia | E78 |
| 4 | Cardiovascular Disease | I20-I25, I63-I69 |
| 5 | COPD (Chronic Obstructive Pulmonary Disease), Emphysema, Chronic Bronchitis | J41-J44, J47 |
| 6 | Diseases of Esophagus, Stomach, and Duodenum | I85, K21-K22, K22.4-K22.7, K23.0-K23.1, K25.4-K25.7, K26.4-K26.7, K27.4-K27.7, K28.4-K28.7, K29.3-K29.9, K31.1-K31.5, Q39-Q40, Z90.3 |
| 7 | Chronic Hepatitis B virus (HBV) infection | B18.0–B18.1 |
| 8 | Chronic Hepatitis C virus (HCV) infection | B18.2 |
| 9 | Chronic kidney disease (CKD) | I12.0–I13.9, N01–N05, N07–N08, N11, N18.3–N18.9, Q60–Q61, Q61.8–Q61.9, Z90.5, Z94.0 |
| 10 | Non–AIDS-related cancers | C00–C45, C47–C52, C54–C81, C86–C95 |
| 11 | Osteoporosis | M80–M82 |
| 12 | Mental health disorders | F20–F48 |
| 13 | Sleep disorders | F51.0–F51.3, G47 |
| 14 | Syphilis | A50–A53 |
| 15 | Human papillomavirus (HPV) infection | A63.0, B07, B97.7, D06, D10.5, D14.1, N87.1–N87.9, N89.0–N89.1, N90.0–N90.1, K62.8 |
| 16 | Metabolic dysfunction–associated steatotic liver disease (MASLD) | K75.8, K76.0 |
| 17 | AIDS-defining cancers | C46, C53, C82–C85, C96 |
| 18 | HIV-associated encephalopathy | B22 |
| 19 | Pneumocystis jirovecii pneumonia (PJP) | B59 |
| 20 | Nontuberculous mycobacterial infections | A31 |
| 21 | Cytomegalovirus (CMV) infection | B25 |
| 22 | Herpes simplex virus (HSV) infection | B00 |
| 23 | Varicella-zoster virus (VZV) infection | B01, B02 |
| 24 | Toxoplasmosis | B58 |
| 25 | Fungal infections | B37, B45, B48 |
| 26 | Tuberculosis | A15-A19 |

**Supplemental Table 3. Classification of the 26 conditions by curability, metabolic status, and infectious status**

| Category | Classification | Condition number* |
| --- | --- | --- |
| Curability | Curable condition | 8, 13, 14, 15, 19, 21, 22, 23, 24, 25, 26 |
|  | incurable condition | 1, 2, 3, 4, 5, 6, 7, 9, 10, 11, 12, 16, 17, 18, 20 |
| Metabolic status | Metabolic condition | 1, 2, 3, 11, 16 |
|  | Non-metabolic condition | 4, 5, 6, 7, 8, 9, 10, 12, 13, 14, 15, 17, 18, 19, 20, 21, 22, 23, 24, 25, 26 |
| Infectious status | Infectious condition | 7, 8, 14, 15, 19, 20, 21, 22, 23, 24, 25, 26 |
|  | Non-infectious condition | 1, 2, 3, 4, 5, 6, 9, 10, 11, 12, 13, 16, 17, 18 |

* Condition number refers to the numbered disease entities listed in Supplemental Table 1.

**Supplemental Table 4. Distribution of six comorbidity states and dropout across three waves**

| **Comorbidity states** | **Total, N** | **Proportion, %** | **Comorbidity states (Wave1)** | **Total, N** | **Proportion, %** | **Comorbidity states (Wave2)** | **Total, N** | **Proportion, %** |
| --- | --- | --- | --- | --- | --- | --- | --- | --- |
| Incurable multimorbidity | 456 | 7.66 | Incurable multimorbidity | 1120 | 18.82 | Incurable multimorbidity | 1517 | 25.50 |
| Curable multimorbidity | 46 | 0.77 | Curable multimorbidity | 7 | 0.12 | Curable multimorbidity | 4 | 0.07 |
| Mixed curable–incurable multimorbidity | 1037 | 17.43 | Mixed curable–incurable multimorbidity | 1042 | 17.51 | Mixed curable–incurable multimorbidity | 1007 | 16.92 |
| Single incurable disease | 2739 | 46.03 | Single incurable disease | 3086 | 51.87 | Single incurable disease | 2665 | 44.79 |
| Single curable disease | 208 | 3.50 | Single curable disease | 60 | 1.01 | Single curable disease | 16 | 0.27 |
| No disease | 1464 | 24.61 | No disease | 493 | 8.29 | No disease | 192 | 3.23 |
| Dropout | 0 | 0.00 | Dropout | 142 | 2.39 | Dropout | 549 | 9.23 |

Counts (N) and proportions (%) are shown for Baseline, Wave1, and Wave2. “Incurable/Curable/Mixed curable–incurable” comorbidity states follow the classification in Supplemental Table 3.

**Supplemental Table 5. Comorbidity states distribution and sex difference in different waves**

| **Comorbidity states** | **Male** |  |  | **Female** |  | **Test type** | **p–value** |
| --- | --- | --- | --- | --- | --- | --- | --- |
|  | **Counts** | **Proportion, %** |  | **Counts** | **Proportion, %** |  |  |
| **Baseline** |  |  |  |  |  |  |  |
| Incurable multimorbidity | 411 | 7.95 |  | 45 | 5.78 | Chi-square | <0.05 |
| Curable multimorbidity | 44 | 0.85 |  | 2 | 0.26 | Fisher | 0.08 |
| Mixed curable–incurable multimorbidity | 938 | 18.14 |  | 99 | 12.71 | Chi-square | <0.01 |
| Single incurable disease | 2385 | 46.12 |  | 354 | 45.44 | Chi-square | 0.75 |
| Single curable disease | 196 | 3.79 |  | 12 | 1.54 | Chi-square | <0.01 |
| No disease | 1197 | 23.15 |  | 267 | 34.27 | Chi-square | <0.01 |
| Dropout | 0 | 0.00 |  | 0 | 0.00 | Fisher | 1.00 |
| **Wave1** |  |  |  |  |  |  |  |
| Incurable multimorbidity | 987 | 19.09 |  | 133 | 17.07 | Chi-square | 0.20 |
| Curable multimorbidity | 5 | 0.10 |  | 2 | 0.26 | Fisher | 0.23 |
| Mixed curable–incurable multimorbidity | 956 | 18.49 |  | 86 | 11.04 | Chi-square | <0.01 |
| Single incurable disease | 2640 | 51.05 |  | 446 | 57.25 | Chi-square | <0.01 |
| Single curable disease | 59 | 1.14 |  | 1 | 0.13 | Fisher | <0.01 |
| No disease | 401 | 7.75 |  | 92 | 11.81 | Chi-square | <0.01 |
| Dropout | 123 | 2.38 |  | 19 | 2.44 | Chi-square | 1.00 |
| **Wave2** |  |  |  |  |  |  |  |
| Incurable multimorbidity | 1340 | 25.91 |  | 177 | 22.72 | Chi-square | 0.06 |
| Curable multimorbidity | 3 | 0.10 |  | 1 | 0.13 | Fisher | 0.43 |
| Mixed curable–incurable multimorbidity | 928 | 17.95 |  | 79 | 10.14 | Chi-square | <0.01 |
| Single incurable disease | 2275 | 44.00 |  | 390 | 50.06 | Chi-square | <0.01 |
| Single curable disease | 14 | 0.27 |  | 2 | 0.26 | Fisher | 1.00 |
| No disease | 158 | 3.06 |  | 34 | 4.36 | Chi-square | 0.07 |
| Dropout | 453 | 8.76 |  | 96 | 12.32 | Chi-square | <0.01 |

Counts and proportions by sex across Baseline, Wave1, and Wave2; p–values from chi-square or Fisher’s exact tests.

**Supplemental Table 6. Comorbidity states change over time among male participants**

| **Wave comparison** | **Comorbidity states** | **Test type** | **p**–**value** |
| --- | --- | --- | --- |
| Baseline vs Wave1 | Incurable multimorbidity | Chi-square | <0.01 |
|  | Curable multimorbidity | Chi-square | <0.01 |
|  | Mixed curable–incurable multimorbidity | Chi-square | 0.67 |
|  | Single incurable disease | Chi-square | <0.01 |
|  | Single curable disease | Chi-square | <0.01 |
|  | No disease | Chi-square | <0.01 |
|  | Dropout | Fisher | <0.01 |
| Wave1 vs Wave2 | Incurable multimorbidity | Chi-square | <0.01 |
|  | Curable multimorbidity | Fisher | 0.73 |
|  | Mixed curable–incurable multimorbidity | Chi-square | 0.49 |
|  | Single incurable disease | Chi-square | <0.01 |
|  | Single curable disease | Chi-square | <0.01 |
|  | No disease | Chi-square | <0.01 |
|  | Dropout | Chi-square | <0.01 |
| Baseline vs Wave2 | Incurable multimorbidity | Chi-square | <0.01 |
|  | Curable multimorbidity | Fisher | <0.01 |
|  | Mixed curable–incurable multimorbidity | Chi-square | 0.82 |
|  | Single incurable disease | Chi-square | <0.05 |
|  | Single curable disease | Chi-square | <0.01 |
|  | No disease | Chi-square | <0.01 |
|  | Dropout | Fisher | <0.01 |

p–values are from chi-square tests (or Fisher’s exact tests where appropriate) comparing Baseline vs Wave1 (90 day to 4 years), Wave1 vs Wave2 (4 years to 8 years), and Baseline vs Wave2.

**Supplemental Table 7. Comorbidity states change over time among female participants**

| **Wave comparison** | **Comorbidity states** | **Test type** | **p**–**value** |
| --- | --- | --- | --- |
| Baseline vs Wave1 | Incurable multimorbidity | Chi-square | <0.01 |
|  | Curable multimorbidity | Fisher | 1.00 |
|  | Mixed curable–incurable multimorbidity | Chi-square | 0.35 |
|  | Single incurable disease | Chi-square | <0.01 |
|  | Single curable disease | Fisher | <0.01 |
|  | No disease | Chi-square | <0.01 |
|  | Dropout | Fisher | <0.01 |
| Wave1 vs Wave2 | Incurable multimorbidity | Chi-square | <0.01 |
|  | Curable multimorbidity | Fisher | 1.00 |
|  | Mixed curable–incurable multimorbidity | Chi-square | 0.62 |
|  | Single incurable disease | Chi-square | <0.01 |
|  | Single curable disease | Fisher | 1.00 |
|  | No disease | Chi-square | <0.01 |
|  | Dropout | Chi-square | <0.01 |
| Baseline vs Wave2 | Incurable multimorbidity | Chi-square | <0.01 |
|  | Curable multimorbidity | Fisher | 1.00 |
|  | Mixed curable–incurable multimorbidity | Chi-square | 0.13 |
|  | Single incurable disease | Chi-square | 0.08 |
|  | Single curable disease | Fisher | <0.05 |
|  | No disease | Chi-square | <0.01 |
|  | Dropout | Fisher | <0.01 |

p–values are from chi-square tests (or Fisher’s exact tests where appropriate) for Baseline vs Wave1, Wave1 vs Wave2, and Baseline vs Wave2. Wave definitions as above.

**Supplemental Table 8. Comorbidity states distribution and diagnostic age difference in different waves**

| **Comorbidity states** | **≤ 32 years** |  |  | **> 32 years** |  | **Test type** | **p–value** |
| --- | --- | --- | --- | --- | --- | --- | --- |
|  | **Counts** | **Proportion, %** |  | **Counts** | **Proportion, %** |  |  |
| **Baseline** |  |  |  |  |  |  |  |
| Incurable multimorbidity | 159 | 5.18 |  | 297 | 10.30 | Chi-square | <0.01 |
| Curable multimorbidity | 24 | 0.78 |  | 22 | 0.76 | Chi-square | 1.00 |
| Mixed curable–incurable multimorbidity | 452 | 14.74 |  | 585 | 20.29 | Chi-square | <0.01 |
| Single incurable disease | 1414 | 46.10 |  | 1325 | 45.96 | Chi-square | 0.93 |
| Single curable disease | 139 | 4.53 |  | 69 | 2.39 | Chi-square | <0.01 |
| No disease | 879 | 28.66 |  | 585 | 20.29 | Chi-square | <0.01 |
| Dropout | 0 | 0.00 |  | 0 | 0.00 | Fisher | 1.00 |
| **Wave1** |  |  |  |  |  |  |  |
| Incurable multimorbidity | 394 | 12.85 |  | 726 | 25.18 | Chi-square | <0.01 |
| Curable multimorbidity | 4 | 0.13 |  | 3 | 0.10 | Fisher | 1.00 |
| Mixed curable–incurable multimorbidity | 580 | 18.91 |  | 462 | 16.02 | Chi-square | <0.01 |
| Single incurable disease | 1659 | 54.09 |  | 1427 | 49.50 | Chi-square | <0.01 |
| Single curable disease | 43 | 1.40 |  | 17 | 0.59 | Chi-square | <0.01 |
| No disease | 317 | 10.34 |  | 176 | 6.10 | Chi-square | <0.01 |
| Dropout | 70 | 2.28 |  | 72 | 2.50 | Chi-square | 0.65 |
| **Wave2** |  |  |  |  |  |  |  |
| Incurable multimorbidity | 606 | 19.76 |  | 911 | 31.60 | Chi-square | <0.01 |
| Curable multimorbidity | 2 | 0.07 |  | 2 | 0.07 | Fisher | 1.00 |
| Mixed curable–incurable multimorbidity | 540 | 17.61 |  | 467 | 16.20 | Chi-square | 0.16 |
| Single incurable disease | 1501 | 48.94 |  | 1164 | 40.37 | Chi-square | <0.01 |
| Single curable disease | 11 | 0.36 |  | 5 | 0.17 | Chi-square | 0.26 |
| No disease | 134 | 4.37 |  | 58 | 2.01 | Chi-square | <0.01 |
| Dropout | 273 | 8.90 |  | 276 | 9.57 | Chi-square | 0.40 |

Counts and proportions are presented at Baseline, Wave1, and Wave2 with p–values from chi-square or Fisher’s exact tests as indicated.

**Supplemental Table 9. Comorbidity states change over time among younger (≤32 years) participants**

| **Comparison** | **Comorbidity states** | **Test type** | **p**–**value** |
| --- | --- | --- | --- |
| Baseline vs Wave1 | Incurable multimorbidity | Chi-square | <0.01 |
|  | Curable multimorbidity | Fisher | <0.01 |
|  | Mixed curable–incurable multimorbidity | Chi-square | <0.01 |
|  | Single incurable disease | Chi-square | <0.01 |
|  | Single curable disease | Chi-square | <0.01 |
|  | No disease | Chi-square | <0.01 |
|  | Dropout | Fisher | <0.01 |
| Wave1 vs Wave2 | Incurable multimorbidity | Chi-square | <0.01 |
|  | Curable multimorbidity | Fisher | 0.69 |
|  | Mixed curable–incurable multimorbidity | Chi-square | 0.20 |
|  | Single incurable disease | Chi-square | <0.01 |
|  | Single curable disease | Chi-square | <0.01 |
|  | No disease | Chi-square | <0.01 |
|  | Dropout | Chi-square | <0.01 |
| Baseline vs Wave2 | Incurable multimorbidity | Chi-square | <0.01 |
|  | Curable multimorbidity | Fisher | <0.01 |
|  | Mixed curable–incurable multimorbidity | Chi-square | <0.01 |
|  | Single incurable disease | Chi-square | <0.05 |
|  | Single curable disease | Chi-square | <0.01 |
|  | No disease | Chi-square | <0.01 |
|  | Dropout | Fisher | <0.01 |

p–values are from chi-square tests (or Fisher’s exact tests where appropriate) comparing Baseline vs Wave1, Wave1 vs Wave2, and Baseline vs Wave2.

**Supplemental Table 10. Comorbidity states change over time among older (>32 years) participants**

| **Comparison** | **Comorbidity states** | **Test type** | p–**value** |
| --- | --- | --- | --- |
| Baseline vs Wave1 | Incurable multimorbidity | Chi-square | <0.01 |
|  | Curable multimorbidity | Fisher | <0.01 |
|  | Mixed curable–incurable multimorbidity | Chi-square | <0.01 |
|  | Single incurable disease | Chi-square | <0.01 |
|  | Single curable disease | Chi-square | <0.01 |
|  | No disease | Chi-square | <0.01 |
|  | Dropout | Fisher | <0.01 |
| Wave1 vs Wave2 | Incurable multimorbidity | Chi-square | <0.01 |
|  | Curable multimorbidity | Fisher | 1.00 |
|  | Mixed curable–incurable multimorbidity | Chi-square | 0.89 |
|  | Single incurable disease | Chi-square | <0.01 |
|  | Single curable disease | Chi-square | <0.05 |
|  | No disease | Chi-square | <0.01 |
|  | Dropout | Chi-square | <0.01 |
| Baseline vs Wave2 | Incurable multimorbidity | Chi-square | <0.01 |
|  | Curable multimorbidity | Fisher | <0.01 |
|  | Mixed curable–incurable multimorbidity | Chi-square | <0.01 |
|  | Single incurable disease | Chi-square | <0.01 |
|  | Single curable disease | Chi-square | <0.01 |
|  | No disease | Chi-square | <0.01 |
|  | Dropout | Fisher | <0.01 |

p–values are from chi-square tests (or Fisher’s exact tests where appropriate) comparing Baseline vs Wave1, Wave1 vs Wave2, and Baseline vs Wave2.

**Supplemental Table 11. Schoenfeld residual–based tests of the proportional hazards (PH) assumption for the Cox model**

| **Covariate** | **df** | **χ²** | **p-value** |
| --- | --- | --- | --- |
| Age at diagnosis, years | 3 | 4.14 | 0.25 |
| BMI, kg/m2 | 2 | 1.99 | 0.37 |
| Sex | 1 | 0.32 | 0.57 |
| Marital status | 2 | 0.31 | 0.85 |
| Baseline CD4 count, cells/μL | 3 | 1.07 | 0.79 |
| PLT, 10^9/L | 2 | 0.47 | 0.79 |
| LDL-C, mg/dL | 1 | 1.11 | 0.29 |
| HDL-C, mg/dL | 1 | 0.06 | 0.81 |
| FPG, mg/dL | 1 | 0.02 | 0.89 |
| Time from HIV diagnosis to ART initiation, months | 2 | 0.58 | 0.75 |
| Baseline ART regimen | 1 | 2.46 | 0.12 |
| ALT, U/L | 2 | 5.68 | 0.06 |
| eGFR, mL/min/1.73m^2^ | 2 | 3.24 | 0.2 |
| Route of HIV transmission | 3 | 1.8 | 0.62 |
| Opportunistic infection | 1 | 1.39 | 0.24 |
| Smoking | 1 | 0.76 | 0.38 |
| Drinking | 1 | 0.55 | 0.46 |
| Global test | 29 | 24.68 | 0.69 |

Values are from the Schoenfeld residual test (cox.zph). For each covariate, the table reports degrees of freedom (df), the chi-square statistic (χ²), and the corresponding p-value. A p-value < 0.05 indicates evidence against the proportional hazards assumption for that covariate. The “Global test” evaluates the PH assumption for the model overall. BMI, body mass index; PLT, platelet count; LDL-C, low-density lipoprotein cholesterol; HDL-C, high-density lipoprotein cholesterol; FPG, fasting plasma glucose; ART, antiretroviral therapy; ALT, alanine aminotransferase; eGFR, estimated glomerular filtration rate.

**Supplemental Figure 1. Sensitivity analysis of Cox regression on metabolic multimorbidity using an extended 6-month landmark after ART initiation**


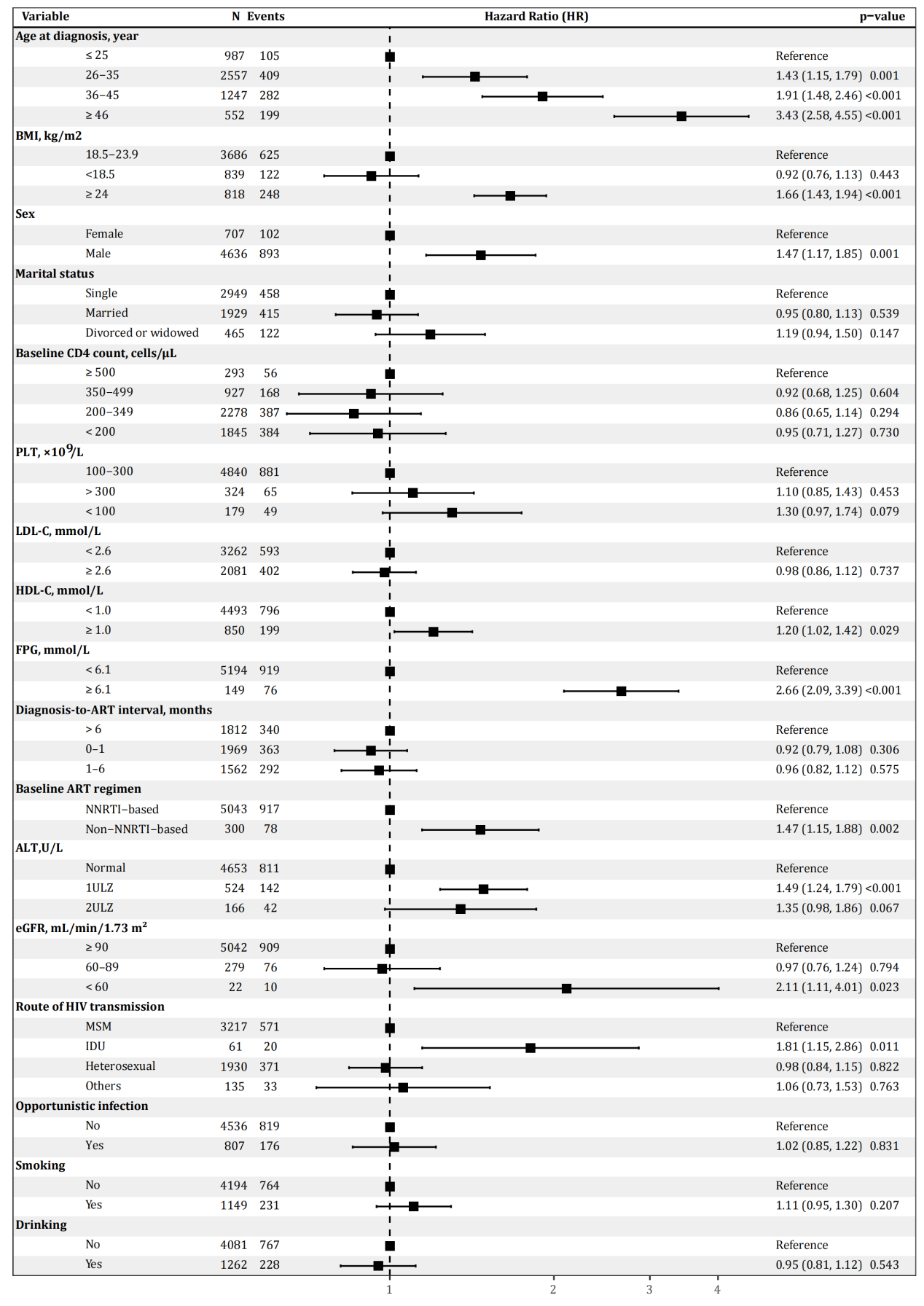


Forest plot from the sensitivity analysis using Cox regression. ART initiation within 6 months was used as the baseline definition for metabolic multimorbidity outcomes. Estimates are adjusted for the same covariates as in the primary Cox model (Figure 5).

**Supplemental Figure 2. Sensitivity analysis of Cox regression on metabolic multimorbidity restricted to Wave1**

**
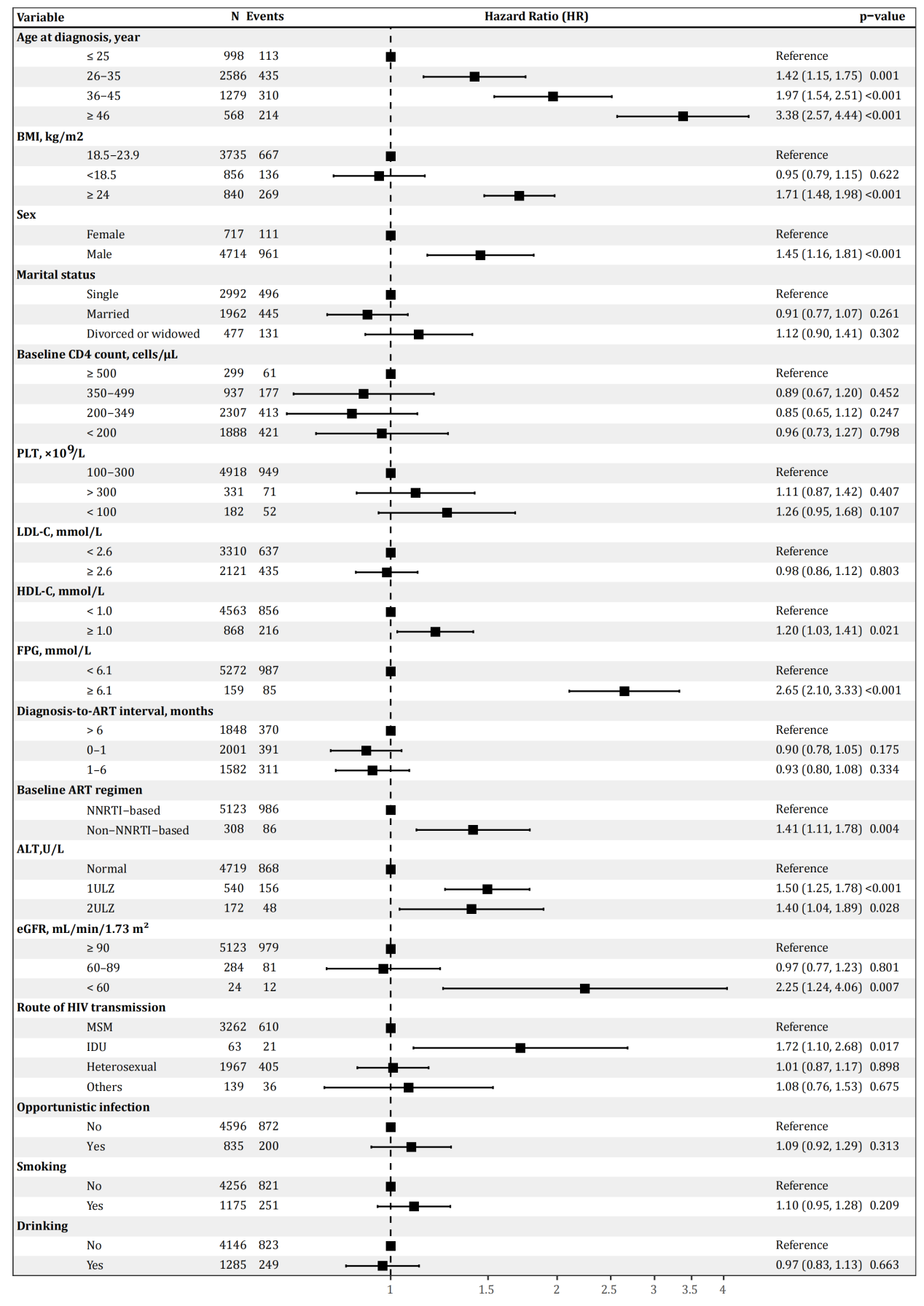
**

Forest plot from the sensitivity analysis using Cox regression. Wave1 (day 90 to 4 years) was used as the restricted follow-up window for metabolic multimorbidity outcomes. Estimates are adjusted for the same covariates as in the primary Cox model (Figure 5).

**Supplemental Figure 3. Sensitivity analysis of logistic regression on mixed infectious–non-infectious multimorbidity restricted to Wave1**


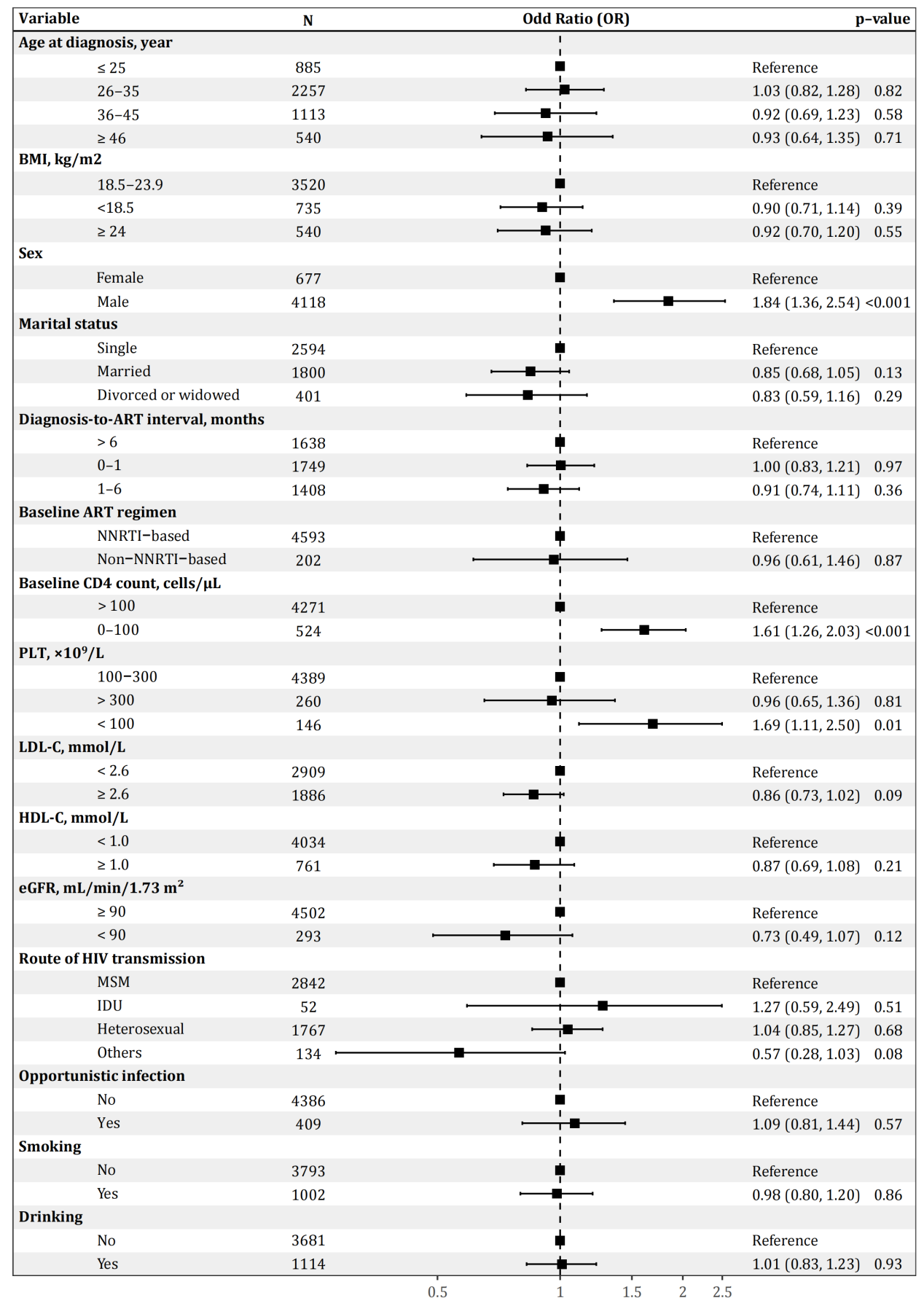


Forest plot from the sensitivity analysis using logistic regression. Wave1 (day 90 to 4 years) was used as the restricted follow-up window for mixed infectious–non-infectious multimorbidity outcomes. Estimates are adjusted for the same covariates as in the primary logistic model (Figure 6).
